# Fisher discriminant model based on LASSO logistic regression for computed tomography imaging diagnosis of pelvic rhabdomyosarcoma in children

**DOI:** 10.1038/s41598-022-20051-8

**Published:** 2022-09-17

**Authors:** Lu Tian, Xiaomeng Li, Helin Zheng, Longlun Wang, Yong Qin, Jinhua Cai

**Affiliations:** 1 Department of Radiology, Children’s Hospital, Chongqing Medical University and Ministry of Education Key Laboratory of Child Development and Disorders, Chongqing International Science and Technology Cooperation Center for Child Development and Disorders and Key Laboratory of Pediatrics in Chongqing, Chongqing, 400014 China; 2 Children’s Hospital of Chongqing Medical University, Chongqing, China

**Keywords:** Oncology, Cancer imaging, Paediatric research

## Abstract

Computed tomography (CT) has been widely used for the diagnosis of pelvic rhabdomyosarcoma (RMS) in children. However, it is difficult to differentiate pelvic RMS from other pelvic malignancies. This study aimed to analyze and select CT features by using least absolute shrinkage and selection operator (LASSO) logistic regression and established a Fisher discriminant analysis (FDA) model for the quantitative diagnosis of pediatric pelvic RMS. A total of 121 pediatric patients who were diagnosed with pelvic neoplasms were included in this study. The patients were assigned to an RMS group (n = 36) and a non-RMS group (n = 85) according to the pathological results. LASSO logistic regression was used to select characteristic features, and an FDA model was constructed for quantitative diagnosis. Leave-one-out cross-validation and receiver operating characteristic (ROC) curve analysis were used to evaluate the diagnostic ability of the FDA model. Six characteristic variables were selected by LASSO logistic regression, all of which were CT morphological features. Using these CT features, the following diagnostic models were established: (RMS group)${G}_{1}=-14.283+6.613{x}_{1}+5.333{x}_{2}+5.753{x}_{3}+12.361{x}_{4}+8.095{x}_{5}-0.715{x}_{6}$; (Non-RMS group)${G}_{2}=-2.008+3.539{x}_{1}+1.080{x}_{2}+1.154{x}_{3}+2.307{x}_{4}+1.656{x}_{5}+1.380{x}_{6}$, where ${x}_{1}$, ${x}_{2}$, … and ${x}_{6}$ are lower than normal muscle density (1 = yes; 0 = no), multinodular fusion (1 = yes; 0 = no), enhancement at surrounding blood vessels (1 = yes; 0 = no), heterogeneous progressive centripetal enhancement (1 = yes; 0 = no), ring enhancement (1 = yes; 0 = no), and hemorrhage (1 = yes; 0 = no), respectively. The calculated area under the ROC curve (AUC) of the model was 0.992 (0.982–1.000), with a sensitivity of 94.4%, a specificity of 96.5%, and an accuracy of 95.9%. The calculated sensitivity, specificity and accuracy values were consistent with those from cross-validation. An FDA model based on the CT morphological features of pelvic RMS was established and could provide an easy and efficient method for the diagnosis and differential diagnosis of pelvic RMS in children.

## Introduction

Rhabdomyosarcoma (RMS) is a soft tissue sarcoma that accounts for approximately 4.5% of pediatric cancer cases and is characterized by a high degree of malignancy, infiltration of adjacent tissues, and early lymph node and distant metastases^1^. The pelvic cavity is one of the most common sites, second only to the head and neck^2^. Accurate preoperative diagnosis is very important to provide appropriate planning for surgery and could lead to a better prognostic outcome. In recent years, medical imaging technology has made great progress and is playing an increasingly important role in the diagnosis of tumours. Of many imaging modalities, computed tomography (CT) has the advantages of noninvasiveness, high spatial and density resolution, and high scanning speed and has been widely applied for the assessment of tumours in the abdomen, pelvis and thorax. CT images allow radiologists not only to determine the extent of tumours but also to determine the absence or presence of bony destruction, calcification, haemorrhage and/or metastases^3^, However, in fact, during the course of routine radiology diagnosis, because RMS has the general radiological appearance of soft tissue tumors, it is difficult to distinguish it from other pelvic soft tissue malignancies. However, to our knowledge, there are currently few literature reports on the CT features of pelvic RMS^4–9^, and some reports have indicated that the CT findings of pelvic RMS lack specificity^10–12^. Therefore, it is imperative to investigate the CT features of pelvic RMS and establish an accurate diagnostic method for pelvic RMS in children.

Least absolute shrinkage and selection operator (LASSO), first proposed by Robert Tibshirani in 1996, is a regression analysis method to reduce the dimensionality of data. Serving as a regularized estimation, LASSO can shrink the coefficients of variables and force certain regression coefficients to 0 by constructing a penalty function, so it is often adopted for variable selection before establishing prediction and diagnostic models^13,14^. Fisher discriminant analysis (FDA) is a classic method for identifying linear functions of variables to distinguish samples of different groups^15^. It was proposed by Fisher in 1936 and serves as a dimensionality reduction method that finds a linear combination of features that maximizes the between-class differences and minimizes the within-class variation^16^. FDA has been widely used in many fields, including medical research^17^. To date, a variety of studies have applied FDA to predict, diagnose or identify diseases^18–20^. A study by Ni et al.^18^ applied FDA to predict clinicopathological subtypes of breast cancer based on the radiological features of diffusion-weighted magnetic resonance imaging (MRI) and suggested that FDA was a promising method for predicting clinicopathological subtypes of breast cancer. In a report by Zou et al.^19^, FDA was applied to classify autism spectrum disorders (ASDs) based on folate-related metabolic markers, and the results showed that the FDA model could effectively distinguish ASD patients from healthy controls. Hao et al.^20^ used FDA to establish a discriminant formula to distinguish patients with gastric cancer and colorectal cancer from healthy controls and achieved good results.

In this study, we used LASSO logistic regression to evaluate and select valuable CT features. Based on these features, an FDA model for pelvic RMS diagnosis was established, and the diagnostic accuracy was validated. Our aim was to develop a simple and accurate diagnostic method for the quantitative diagnosis of pelvic RMS by means of mathematical statistics.

We present the following article in accordance with the Tripod Checklist.

## Materials and methods

Clinical data and pathological results were obtained from patients' medical records. Imaging data were retrieved from our picture archiving and communication system.

### Study subjects

A total of 121 pediatric patients who underwent abdominal contrast-enhanced CT scans and were diagnosed with pelvic tumors from January 2013 to January 2021 were included in this study. According to the pathological results, these patients were divided into RMS and non-RMS groups. The RMS group included 36 patients (14 males, 22 females) with ages ranging from 1 month to 16 years, and the non-RMS group included 85 patients (24 males, 61 females) with ages ranging from 1 month to 15 years. Those in the non-RMS group suffered from a variety of malignant tumors, including 19 patients with yolk sac tumors, 18 patients with malignant teratoma, 13 patients with neuroblastoma, 9 patients with lymphoma, 5 patients with Ewing sarcoma and 21 patients with other types of malignant reproductive tumors.

### CT imaging and feature extraction

All pediatric patients in our study underwent pelvic plain and contrast-enhanced CT examinations on a 256-slice spiral CT system (Philips Brilliance iCT, Philips, Netherlands) with a low tube voltage of 80–100 kV, a low tube current of 100–200 mA (the current varied during the acquisition and according to the child’s body weight), a rotation time of 0.4 s, a pitch of 0.925, a collimation of 128*0.625 mm, a slice thickness of 5.0 mm and a reconstruction layer thickness of 1 mm. To obtain arterial and venous phase contrast-enhanced images, dual-phase dynamic contrast-enhanced CT was performed at 30 and 65 s, respectively, after intravenous injection of the contrast agent (Omnipaque, 350 mg/mL, Amersham Healthcare, Shanghai, China). The contrast agent was administered at a dose of 2 mL/kg body weight, with a maximum of 80 mL.

During the course of routine radiology diagnosis, for tumor lesions, we mainly need to pay attention to their morphology, density, margin, enhancement mode and metastasis. Therefore, our study mainly selected image features from these five aspects to explore the differences between the two groups (RMS group and non-group). According to previous literature and diagnostic experience. The following CT features of the tumors were evaluated: (a) morphology (multinodular fusion/lobulated/round/orbicular); (b) density (lower or higher than normal muscle density/calcification/hemorrhage/necrosis); (c) margin (clear or unclear); (d) contrast enhancement modes (surrounding blood vessels/homogeneous progressive centripetal enhancement/ring enhancement/grape cluster reinforcement); (e) metastasis (lymphatic metastasis/bone erosion). The evaluation criteria for the above relatively special CT features are as follows: (1) multinodular fusion: in the CT images, multiple nodules of different sizes were observed in the pelvic cavity. Some of the nodules were fused together and fused into a lobulated mass^21^; (2) surrounding blood vessels: multiple strip-like and punctate vascular shadows can be seen in the mass on CT enhanced scan images^21^; (3) homogeneous progressive centripetal enhancement: on dynamic contrast-enhanced CT images, the mass can be seen with peripheral annular inhomogeneous enhancement in the arterial phase, and gradually centripetal inhomogeneous enhancement in the venous and delayed phases^21^; (4) grape cluster reinforcement: when the mass is in a hollow structure (vaginal or bladder, etc.), a mass like a grape cluster will appear on the CT-enhanced image^22^; (5) lymph node metastasis: cervical lymph nodes I, II and inguinal lymph nodes short diameter ≥ 1.5 cm, other cervical lymph nodes short diameter ≥ 1 cm, or the degree of enhancement was significantly higher than muscle tissue^23^.

Prospective evaluation of the CT images for each patient was independently performed by two abdominal radiologists with 10–20 years of experience. The abdominal radiologists were blinded to patient characteristics and histologic results and evaluated the morphology, density, margin, enhancement modes and metastasis of the tumors. In case of disagreement between the two radiologists, a consensus was reached with a third senior abdominal radiologist and two other abdominal radiologists.

### Statistical analysis

The patients were divided into an RMS group and a non-RMS group according to the pathological results. For quantitative variables, continuous variables that followed a normal distribution are described as the mean and standard deviation (SD), and a parametric t test was used to determine the statistical significance between the two groups. Otherwise, the variables are described as medians and interquartile ranges (IQRs), and a nonparametric Mann–Whitney U test was used for comparisons between the two groups. Categorical variables are expressed as the number of patients and respective percentage, and the χ^2^ test or Fisher's exact test was used to compare the rates.

LASSO logistic regression was used to select the optimal characteristic features for diagnosing RMS from the basic and CT morphological features of the patients. The penalty parameter λ was optimized, and the resulting nonzero coefficient variables in the model were selected as the diagnostic variables. Based on these findings, FDA was established as a quantitative diagnostic model of pediatric pelvic RMS. The diagnostic ability of this model was evaluated by the receiver operating characteristic (ROC) curve. Additionally, the cumulative diagnostic ability of the features was analyzed.

A two-tailed P < 0.05 was considered to be statistically significant. All statistical analyses were performed using R 3.6.1 software and SPSS 23.0 software (IBM, Armonk, New York, USA).

### Establishing the model

FDA is a classical approach to identify a linear function of variables to distinguish samples from different groups as much as possible^13^. In our study, patients with RMS and without RMS were set as the two groups: ${G}_{1}$ (RMS group) and ${G}_{2}$ (non-RMS group). A total of 6 CT features (${x}_{i}$) were used as diagnostic variables to establish a linear discriminant function:

$${G}_{i}={\sum }_{j=1}^{6}{c}_{j}{x}_{ij} \begin{array}{c}i=\mathrm{1,2}\end{array}$$

By using the discriminant rule, the result of the examination was found to belong to ${G}_{1}$ or ${G}_{2}$.

(1) Raw data matrix: Two matrices (${W}^{1},{W}^{2}$) were constructed for ${G}_{1}$ and, ${G}_{2}$, with CT features as the column vectors and pediatric patients as the row vectors.

Data matrix of ${G}_{1}$:

$${W}^{1}=\left[\begin{array}{cccc}{1}& {1}& \text{...}& {1}\\ {2}& {0}& \text{...}& {1}\\ \text{...}& \text{...}& \text{...}& \text{...}\\ {36}& {1}& \text{...}& {0}\end{array}\right]$$

Data matrix of ${G}_{2}$:

$${W}^{2}=\left[\begin{array}{cccc}{37}& {0}& \text{...}& {1}\\ {51}& {1}& \text{...}& {0}\\ \text{...}& \text{...}& \text{...}& \text{...}\\ {121}& {0}& \text{...}& {1}\end{array}\right]$$

(2) The mean column distributions of matrices *W*^*1*^ and *W*^*2*^ are as follows:

$$\overline{{{x}_{j}}^{1}}=\frac{1}{36}{\sum }_{i=1}^{36}{{X}_{ij}}^{1} \begin{array}{c}j=\mathrm{1,2}\cdots ,6\end{array}$$

$$\overline{{{x}_{j}}^{2}}=\frac{1}{85}{\sum }_{i=1}^{85}{{X}_{ij}}^{2} \begin{array}{c}j=\mathrm{1,2}\cdots ,6\end{array}$$

(3) The coefficients ${c}_{i}$ were calculated using the differential calculus method.

(4) The discriminant function is defined as:

$${G}_{i}={c}_{1}{x}_{1}+{c}_{2}{x}_{2}+{c}_{3}{x}_{3}+{c}_{4}{x}_{4}+{c}_{5}{x}_{5}+{c}_{6}{x}_{6}$$

(5) The discriminant values represented by ${G}_{1}$ and ${G}_{2}$ were calculated.

$$\overline{{G}_{1}}={\sum }_{i=1}^{36}{c}_{i}\overline{{x}_{i}^{1}}$$

$$\overline{{G}_{2}}={\sum }_{i=1}^{85}{c}_{i}\overline{{x}_{i}^{2}}$$

(6) The value of the Fisher discrimination function at the centroids was obtained as follows:

$${G}_{0}=\frac{36\overline{{G}_{1}}+85\overline{{G}_{2} }}{121}$$

(7) The above calculation was conducted using SPSS software with the following discriminant rule: If $\overline{{G}_{1}}$>${G}_{0}$, the sample of $G$ belongs to ${G}_{1}$, which means that the sample belongs to the RMS group. Otherwise, the sample belongs to ${G}_{2}$; that is, the sample belongs to the non-RMS group.

(8) The resulting discriminant functions for classification were also calculated using SPSS.

$${G}_{1}={c}_{11}{x}_{1}+{c}_{21}{x}_{2}+{c}_{31}{x}_{3}+{c}_{41}{x}_{4}+{c}_{51}{x}_{5}+{c}_{61}{x}_{6}(\mathrm{RMS group})$$

$${G}_{2}={c}_{12}{x}_{1}+{c}_{22}{x}_{2}+{c}_{32}{x}_{3}+{c}_{42}{x}_{4}+{c}_{52}{x}_{5}+{c}_{62}{x}_{6} (\mathrm{non}-\mathrm{RMS group})$$

The values of ${G}_{1}$ and ${G}_{2}$ were calculated by substituting the CT features into the function. By comparing the ${G}_{1}$ and ${G}_{2}$ values, the subjects were classified according to the following principle: if ${G}_{1}$ > ${G}_{2}$, the subjects were classified into the RMS group; if ${G}_{1}$< ${G}_{2}$, the subjects were classified into the non-RMS group.

Notations:

${G}_{i}$: population of disease, $i=1, 2$;

${W}^{i}$: data matrix of ${G}_{i}$;

${c}_{i}$: coefficients of Fisher's discriminant function, $i=0, 1, 2,\cdots , 6$;

${G}_{0}$: values of Fisher discrimination function at centroids;

${x}_{ij}$: content of the $j$ CT feature in the $i$ patient.

### Model validation

Leave-one-out cross-validation, in which each respective case is classified using all cases other than that case for deriving the classification formula, was used to validate the accuracy of the model. In addition, ROC curves were used to validate the accuracy of the model, where an area under the ROC curve (AUC) between 0.5 and 0.7 represented a low diagnostic value, that between 0.7 and 0.9 represented a medium diagnostic value, and that more than 0.9 represented a high diagnostic value^24^.

### Ethical statement

The study was conducted in accordance with the Declaration of Helsinki (as revised in 2013). The study was approved by the Institutional Ethics Committee of Children's Hospital Affiliated to Chongqing Medical University and individual consent for this retrospective analysis was waived.

## Results

### Basic and CT morphological features of the patients

RMS (36/121, 29.7%) was the most common pelvic malignant tumor in our study, followed by yolk sac tumor (19/121, 15.7%), malignant teratoma (18/121, 14.9%), neuroblastoma (13/121, 10.7%), lymphoma (9/121, 7.4%) and Ewing sarcoma (5/121, 4.1%). There was no significant difference in age or sex between the two groups (both P > 0.05). A comparison of the basic and CT morphological characteristics between the two groups is shown in Table 1.

**Table 1:** Comparison of the basic and CT morphological characteristics between the RMS group and the non-RMS group.

	RMS group (n = 36)	Non-RMS group (n = 85)	Statistic	P value
**Baseline**				
Age (year) Q (Q1–Q3)	2.5 (1–6.5)	6 (1–9)	1.767 ^a^	0.077
Sex (male/female)	14/22	24/61	1.332 ^b^	0.248
**Density N (%)**				
Lower than normal muscle density	35 (97.2)	51 (60.4)	17.043 ^b^	< 0.001
Calcification	1 (2.8)	23 (27.1)	9.377 ^b^	0.002
Hemorrhage	5 (13.9)	28 (32.9)	4.628 ^b^	0.031
Necrosis	35 (97.2)	71 (83.5)		0.066
**Shape N (%)**				
Multinodular fusion	23 (63.9)	10 (11.8)	34.641 ^b^	< 0.001
Lobulated	28 (77.8)	44 (51.8)	7.102 ^b^	0.008
Round/orbicular	6 (16.7)	22 (25.9)	1.208 ^b^	0.272
**Margin N (%)**				
Unclear	8 (22.2)	26 (30.6)	0.876 ^b^	0.349
**Enhancement feature N (%)**				
Surrounding blood vessels	29 (80.6)	16 (18.8)	41.257 ^b^	< 0.001
Heterogeneous progressive centripetal enhancement	31 (86.1)	10 (11.8)	62.395 ^b^	< 0.001
Ring enhancement	5 (13.9)	6 (7.1)	c	0.300
Grape cluster reinforcement	0 (0.0)	71 (0.0)	–	–
**Metastasis N (%)**				
Lymphatic metastasis	12 (33.3)	14 (16.5)	4.263 ^b^	0.039
Bone erosion	2 (5.6)	5 (5.9)	c	1.000

Q is the median age, Q1–Q3 are 25–75% quantiles.

*CT* computed tomography, *RMS* rhabdomyosarcoma.

^a^Using the M–U test.

^b^Using the Chi-square test.

^c^Using Fisher's exact probability test.

### Selection of diagnostic features

**Figure 1:**
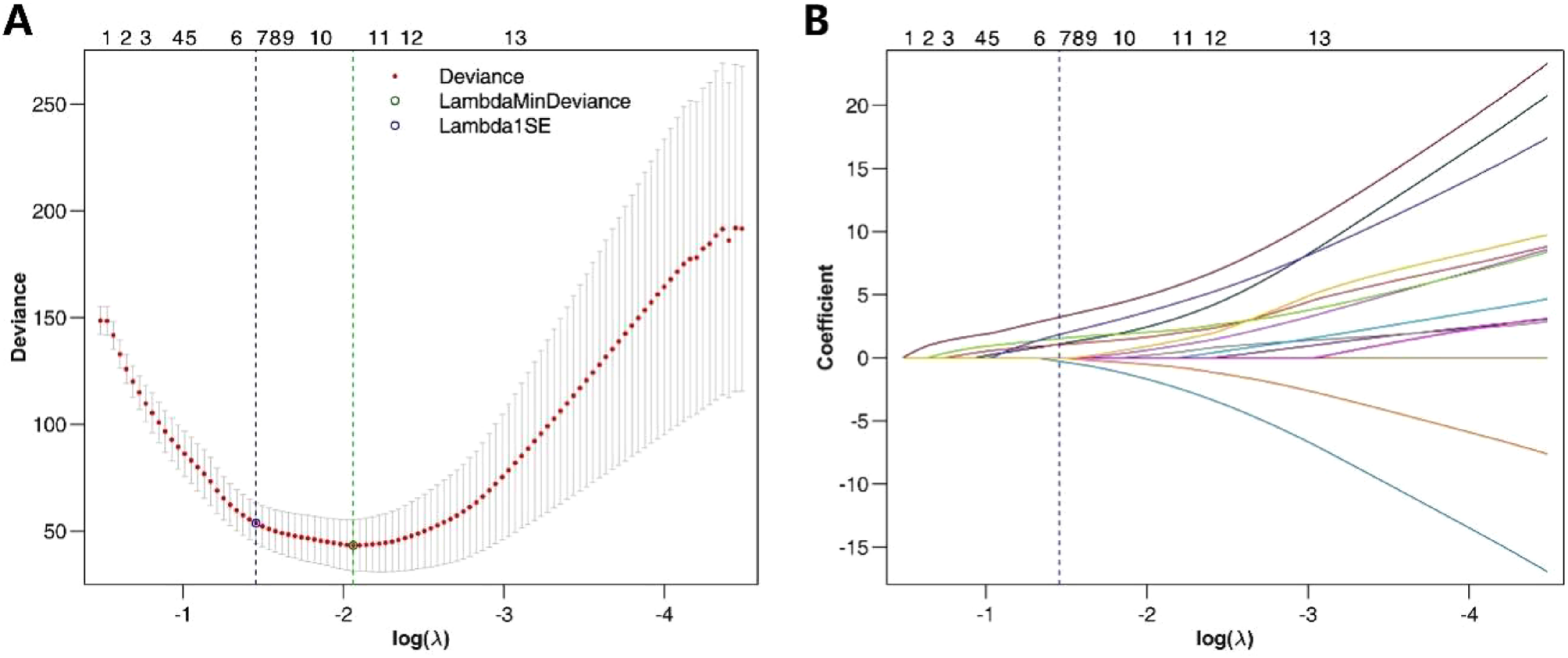
LASSO logistic regression plot. (**A**) Plot of partial likelihood deviance; (**B**) plot of LASSO coefficient profiles. Each colored curve represents the LASSO coefficient profile of a feature against the log (λ) sequence.

LASSO logistic regression included a total of 16 basic factors (2/16) and CT morphological characteristics (14/16) of all patients to select potential diagnostic factors. The LASSO regression partial likelihood deviation and coefficient profiles against log (λ) are shown in Fig. 1. Six variables with nonzero coefficients were selected, all of which were CT morphological features, including lower than normal muscle density, multinodular fusion, enhancement at surrounding blood vessels, heterogeneous progressive centripetal enhancement, ring enhancement and hemorrhage. These variables then served as the preferred features for the diagnosis of RMS.

### Construction of the diagnostic model

Through the construction of the fisher discriminant model, the discriminant functions of RMS and non-RMS were obtained as follows:

$${G}_{1}=-14.283+6.613{x}_{1}+5.333{x}_{2}+5.753{x}_{3}+12.361{x}_{4}+8.095{x}_{5}-0.715{x}_{6};$$

$${G}_{2}=-2.008+3.539{x}_{1}+1.080{x}_{2}+1.154{x}_{3}+2.307{x}_{4}+1.656{x}_{5}+1.380{x}_{6};$$

where ${x}_{1}$, ${x}_{2}$, … and ${x}_{6}$ represent lower than normal muscle density (1 = yes; 0 = no), multinodular fusion (1 = yes; 0 = no), enhancement at surrounding blood vessels (1 = yes; 0 = no), heterogeneous progressive centripetal enhancement (1 = yes; 0 = no), ring enhancement (1 = yes; 0 = no), and hemorrhage (1 = yes; 0 = no), respectively. Wilks’ lambda of the model = 0.245, χ^2^ = 163.237, df = 6, and P < 0.001. The function value at the model centroid was 2.676 in the RMS group and − 1.133 in the non-RMS group, and the critical value of the discriminant function was G0 = 0.800.

### Accuracy and cross-validation of the diagnostic model

**Figure 2:**
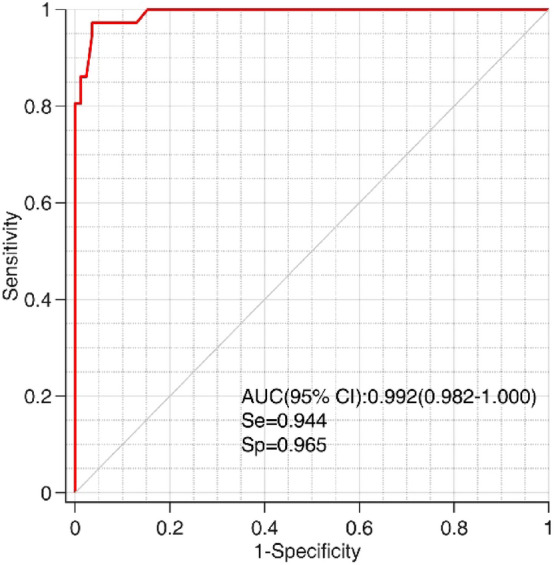
Receiver operating characteristic (ROC) curve of the quantitative diagnostic model for pelvic RMS in children using Fisher discriminant analysis.

The ROC curve was used to verify the accuracy of the diagnostic model (Fig. 2). The AUC of the FDA score in this study was 0.992 (95% confidence interval: 0.982–1.000; sensitivity: 94.4%, specificity: 96.5%), and the accuracy of the model was 95.9%. The result of the cross-validation also showed an accuracy rate of 95.9%. The model correctly classified 34 patients (94.4%) in the RMS group, with a misclassification rate of 5.6%, and 82 patients (96.5%) in the non-RMS group, with a misclassification rate of 3.5% (Table 2).

**Table 2:** Results of the Fisher model and cross-validation.

	Predicted (n, %)	
	RMS	Non-RMS
**Fisher model**		
RMS group	33 (91.7)	3 (8.3)
Non-RMS group	2 (2.4)	83 (97.6)
**Cross-validation**		
RMS group	33 (91.7)	3 (8.3)
Non-RMS group	2 (2.4)	83 (97.6)

### Single feature analysis and cumulative FDA

**Figure 3:**
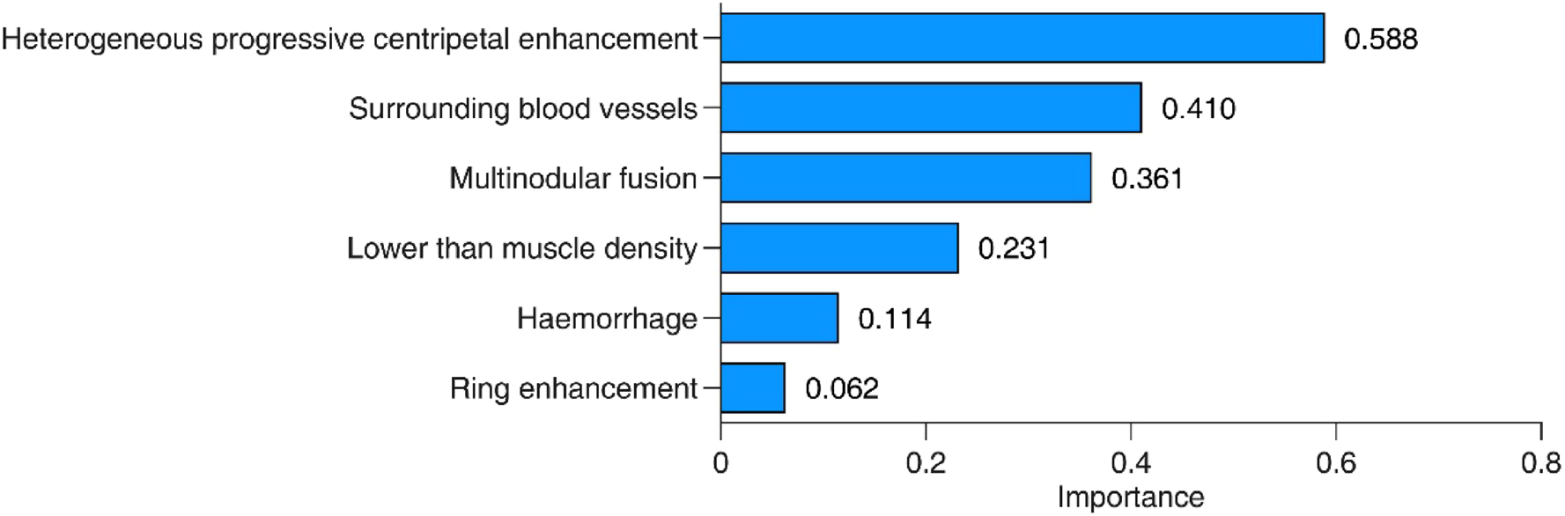
Importance of fisher discriminant model features.

The 6 selected CT morphological features were ordered by their importance according to the Fisher discriminant model as follows: heterogeneous progressive centripetal enhancement, enhancement at surrounding blood vessels, multinodular fusion, lower than normal muscle density, hemorrhage and ring enhancement (Fig. 3). The sensitivity, specificity, AUC and 95% confidence interval of the 6 selected features are shown in Table 3 in order of importance. The sensitivity, specificity and AUC of the FDA score were significantly higher than those of each single CT characteristic feature.

**Table 3:** Sensitivity, specificity, AUC and 95% CI of the single characteristic features.

	Se (95% CI)	Sp (95% CI)	AUC (95% CI)
Heterogeneous progressive centripetal enhancement	0.861 (0.697–0.948)	0.882 (0.790–0.939)	0.872 (0.795–0.948
Enhancement at surrounding blood vessels	0.806 (0.634–0.912)	0.812 (0.709–0.885)	0.809 (0.720–0.898)
Multinodular fusion	0.639 (0.462–0.787)	0.882 (0.790–0.939)	0.761 (0.658–0.864)
Lower than normal muscle density	0.972 (0.838–0.999)	0.400 (0.297–0.512)	0.686 (0.592–0.780)
Hemorrhage	0.861 (0.697–0.948)	0.329 (0.234–0.441)	0.595 (0.489–0.701)
Ring enhancement	0.139 (0.052–0.303)	0.929 (0.847–0.971)	0.534 (0.419–0.649)

*Se* sensitivity, *Sp* specificity, *CI* confidence interval, *AUC* area under the curve.

**Figure 4:**
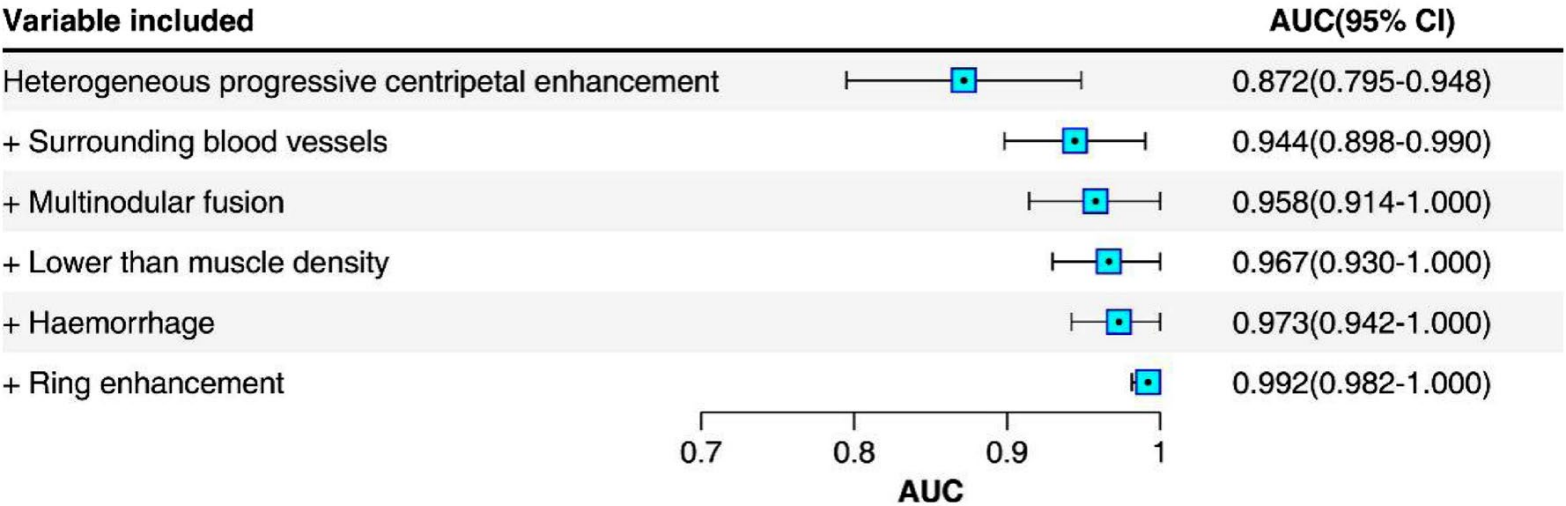
AUC of fisher's discriminant model and its 95% CI accumulated according to the importance of the features. *CI* confidence interval, *AUC* area under the curve.

Next, the feature importance was further evaluated using cumulative fisher discriminant models following the previously determined order of importance (Fig. 4). After the fourth cumulated feature, there was no significant improvement in the diagnostic ability of the discriminant models. Namely, there was no significant difference in the resulting AUCs (all above 0.96) for the first 4 indicators, the first 5 indicators and all 6 indicators (all P > 0.05). This result suggested that using fewer indicators can also accurately diagnose RMS, which is helpful to save resources.

## Discussion

During routine radiological diagnosis, RMS is difficult to distinguish from other pelvic tumors except malignant teratoma, which involves characteristic calcification and fatty composition^10^. Previous studies have shown some CT features of RMS, such as tumoral necrosis, lower density than muscle, clear margin, and susceptibility to lymphatic node or/and bone metastasis^25–37^. However, the specificity and sensitivity of these CT features have not been systematically analyzed due to the limited number of study samples. In this study, we extracted the important CT features of pelvic RMS by using LASSO logistic regression and established a quantitative diagnostic model for pelvic RMS by FDA. This diagnostic model is easy to operate, has a high diagnostic accuracy and can be applied in the diagnosis of pelvic RMS and differentiation from other pelvic tumors in children.

Our study showed that a total of 8 CT features of RMS were significantly different from those of other pelvic malignancies. The differences in these CT features may be related to their pathological characteristics. Studies have shown that pelvic RMS in children is highly invasive and characterized by multicentric growth^35–38^. In the CT images, multiple nodules of different sizes were observed in the pelvic cavity, and some nodules fused together and formed a lobulated mass. Studies also found that some RMS tumor cells were distributed around the blood vessels using microscopes and that the blood vessels were gradually surrounded by tumor cells as they grew^39,40^. This pathological phenomenon is consistent with multiple vascular shadows shown in the contrast-enhanced CT images of pelvic RMS. Moreover, it has been shown that RMS contains abundant fibrous tissue and that the contrast agent gradually permeates into the tumor center over time, persisting in the tumor fibrous tissue for a long time^41,42^. This is possibly why RMS tends to have heterogeneous progressive centripetal enhancement. In addition, the rich mucus in RMS tumors could result in the CT feature of lower than normal muscle density, and rapid tumor growth with nutrient requirements exceeding the vessel supply could contribute to the CT feature of tumoral necrosis.

In this study, we selected 6 CT features with diagnostic value using LASSO logistic regression and established a quantitative diagnostic model of pelvic RMS in children through FDA.

The advantage of this model is that it is simple to operate and easy to implement in routine imaging diagnosis. It is actually a simple mathematical formula that is: (RMS group)${G}_{1}=-14.283+6.613{x}_{1}+5.333{x}_{2}+5.753{x}_{3}+12.361{x}_{4}+8.095{x}_{5}-0.715{x}_{6}$; (Non-RMS group)${G}_{2}=-2.008+3.539{x}_{1}+1.080{x}_{2}+1.154{x}_{3}+2.307{x}_{4}+1.656{x}_{5}+1.380{x}_{6}$, where ${x}_{1}$, ${x}_{2}$, … and ${x}_{6}$ are lower than normal muscle density (1 = yes; 0 = no), multinodular fusion (1 = yes; 0 = no), enhancement at surrounding blood vessels (1 = yes; 0 = no), heterogeneous progressive centripetal enhancement (1 = yes; 0 = no), ring enhancement (1 = yes; 0 = no), and hemorrhage (1 = yes; 0 = no), respectively. After inserting the CT feature values into the model and calculating ${G}_{1}$ and ${G}_{2}$ values. By comparing the ${G}_{1}$ and ${G}_{2}$ values, subjects with unknown classification were classified according to the following principles: if ${G}_{1}$> ${G}_{2}$, the subjects were classified into the RMS group; if ${G}_{1}$< ${G}_{2}$, the subjects were classified into the non-RMS group. Therefore clinicians or radiologists can insert this mathematical formula into an excel document to use it, or make a small software to use it. The ROC curve suggested that the model had a high diagnostic value. In addition, cross-validation showed that the model had a high diagnostic value. Our study also found that the AUC of the overall FDA model was higher than that of each CT characteristic feature. Cumulative FDA was carried out based on the importance of the features. There was no significant improvement in the discrimination performance of the FDA model when using the first 4 features, the first 5 features and all 6 features (in the order of importance: heterogeneous progressive centripetal enhancement, enhancement at surrounding blood vessels, multinodular fusion, lower than normal muscle density, hemorrhage and ring enhancement). Therefore, the number of diagnostic CT features can be reduced to 4, and RMS can still be accurately diagnosed, which is beneficial for saving resources. The establishment of a diagnostic model allows us to go from image diagnosis based on human experiences to quantitative imaging diagnosis, which makes the diagnosis of pelvic RMS simpler and more accurate. In the future, we aim to use the CT diagnostic model of RMS to develop artificial intelligence diagnostic software for clinical practice.

There were some limitations in this study. First, this was a retrospective study, which may have inherent selection bias. Second, the sample size was small. RMS is not a common disease in children; therefore, in future research, we need to further expand the sample size to improve the accuracy of our model. Finally, our study summarized only CT features, and the diagnostic value of MRI for RMS needs to be further studied.

## Conclusions

Our study showed that pelvic RMS in children has some specific CT features. Furthermore, LASSO logistic regression is a reliable method for selecting diagnostic features of RMS. The FDA model based on CT morphological features can accurately diagnose pelvic RMS in children, with promising cross-validation performance. This diagnostic model could provide an easy and efficient method for the diagnosis and differential diagnosis of pelvic RMS in children.

## Data Availability

All data generated or analysed during this study are included in this article and its supplementary information files.

## Abbreviations

FDA: Fisher discriminant model
LASSO: Least absolute shrinkage and selection operator
CT: Computed tomography
RMS: Rhabdomyosarcoma
ROC: Receiver operating characteristic
AUC: Area under the ROC curve
MRI: Magnetic resonance imaging
SD: Standard deviation
